# Diagnostic accuracy of mediastinal width measurement on posteroanterior and anteroposterior chest radiographs in the depiction of acute nontraumatic thoracic aortic dissection

**DOI:** 10.1007/s10140-012-1034-3

**Published:** 2012-03-14

**Authors:** Vincent Lai, Wai Kan Tsang, Wan Chi Chan, Tsz Wai Yeung

**Affiliations:** 1 Department of Diagnostic Radiology, Li Ka Shing Faculty of Medicine, The University of Hong Kong, Queen Mary Hospital, Pokfulam, Hong Kong; 2 Department of Radiology, Tuen Mun Hospital, Tuen Mun, Hong Kong

**Keywords:** Acute aortic dissection, Nontraumatic, Chest radiograph, Computed tomography

## Abstract

We aimed to explore the diagnostic accuracy of various mediastinal measurements in determining acute nontraumatic thoracic aortic dissection with respect to posteroanterior (PA) and anteroposterior (AP) chest radiographs, which had received little attention so far. We retrospectively reviewed 100 patients (50 PA and 50 AP chest radiographs) with confirmed acute thoracic aortic dissection and 120 patients (60 PA and 60 AP chest radiographs) with confirmed normal aorta. Those who had prior history of trauma or aortic disease were excluded. The maximal mediastinal width (MW) and maximal left mediastinal width (LMW) were measured by two independent radiologists and the mediastinal width ratio (MWR) was calculated. Statistical analysis was then performed with independent sample *t* test. PA projection was significantly more accurate than AP projection, achieving higher sensitivity and specificity. LMW and MW were the most powerful parameters on PA and AP chest radiographs, respectively. The optimal cutoff levels were LMW = 4.95 cm (sensitivity, 90 %; specificity, 90 %) and MW = 7.45 cm (sensitivity, 90 %; specificity, 88.3 %) for PA projection and LMW = 5.45 cm (sensitivity, 76 %; specificity, 65 %) and MW = 8.65 cm (sensitivity, 72 %; specificity, 80 %) for AP projection. MWR was found less useful and less reliable. The use of LMW alone in PA film would allow more accurate prediction of aortic dissection. PA chest radiograph has a higher diagnostic accuracy when compared with AP chest radiograph, with negative PA chest radiograph showing less probability for aortic dissection. Lower threshold for proceeding to computed tomography aortogram is recommended however, especially in the elderly and patients with widened mediastinum on AP chest radiograph.

## Introduction

Thoracic aortic injury is a potential lethal entity. Although it can be accurately assessed by cross-sectional imaging such as multidetector computed tomography (CT) or transesophageal dissection nowadays [1, 2], chest radiograph is often the initial imaging modality performed when clinical suspicion of aortic dissection is raised. Mediastinal width (MW) has been the most commonly used criterion with a quoted cutoff value ranging from 7.3 to 9.4 cm [3–7]. Despite its relatively poor diagnostic accuracy [8–10] with significantly low specificity, it had been found to be the most powerful radiographic tool and improved specificity had been observed with the use of left mediastinal width (LMW) and mediastinal width ratio (MWR) [11]. Nevertheless, results from past literatures mainly focused on traumatic aortic rupture [12] but seldom on nontraumatic thoracic aortic rupture or aortic dissection which is far more common but often less emphasized with little attention received. In addition, the impact of posteroanterior (PA) and anteroposterior (AP) chest radiograph on mediastinal measurement was not well established and studied. Hence, in our study, we aimed to explore the accuracy and diagnostic power of various mediastinal measurements in determining acute nontraumatic thoracic aortic dissection with respect to PA and AP chest radiographs, from which to derive the desire cutoff values.

## Materials and methods

### Patient selection

We retrospectively reviewed all cases of suspected or confirmed acute aortic dissection that had both chest radiograph and CT scan of the thorax performed during the same emergency admission over a period of 6 years from 2005 to 2010. Exclusion criteria include those who had known history of thoracic aortic aneurysm, aortic dissection, mediastinal disease, pericardial effusion, cardiomyopathy, collapsed consolidation of the lungs, central lung disease obscuring the mediastinum, prior thoracic surgery, and prior history of trauma. Those who did not present with acute chest pain to the emergency department but with CT-confirmed aortic dissection were excluded to avoid inclusion of chronic aortic dissection cases which might induce interpretation error. Those who had a chest radiograph taken at more than 1 day apart from the CT scan or with significant rotation were also excluded. Through our computer database system, a total of 100 patients (mean, 66.9 ± 16.7 years; range from 24 to 91 years old) matching the above criteria were included, in which 50 patients had a PA chest radiograph taken, while the remaining 50 patients had an AP chest radiograph taken. From the data bank, we also identified and recruited 120 patients (mean, 65.8 ± 15.3 years; range from 28 to 93 years old) with CT-confirmed normal aorta as a control group to match the dissection group, in which 60 patients had a PA chest radiograph taken, while the remaining 60 patients had an AP chest radiograph taken. They were typically retrieved from the initially suspected dissection cases which subsequently proved to be normal by CT scan.

### Imaging techniques

All chest radiographs were taken in a standard standing PA manner or supine/sitting AP manner. The standard focal film distance (FFD) employed were standing PA, 183 cm FFD; sitting AP, 135 cm FFD; supine AP, 102 cm FFD. For the 50 AP chest radiographs in the dissection group, 23 were of sitting film, while the remaining 27 were taken in the supine manner. For the 60 AP chest radiographs in the normal group, 38 were of sitting film, while the remaining 22 were taken in the supine manner. However, no statistical significance was found between the measured values from sitting and supine AP films in both normal and dissection groups. All CT aortograms were performed by our multidetector CT machine (16-head, Brillance 16, Philips). Scanning range was from lung apex to pubic symphysis in all patients. Imaging was performed in the noncontrast phase initially and subsequently during the arterial phase after a bolus (2 mL/kg of body weight; maximum dose, 80 mL) of nonionic iodinated contrast (Omnipaque 300) administered intravenously by mechanical injector at a rate of 4 mL/s. All CT scans were not cardiac-gated since this was considered not appropriate in the emergency setting. The raw images acquired were then reconstructed for interpretation.

### Imaging measurement

**Fig. 1:**
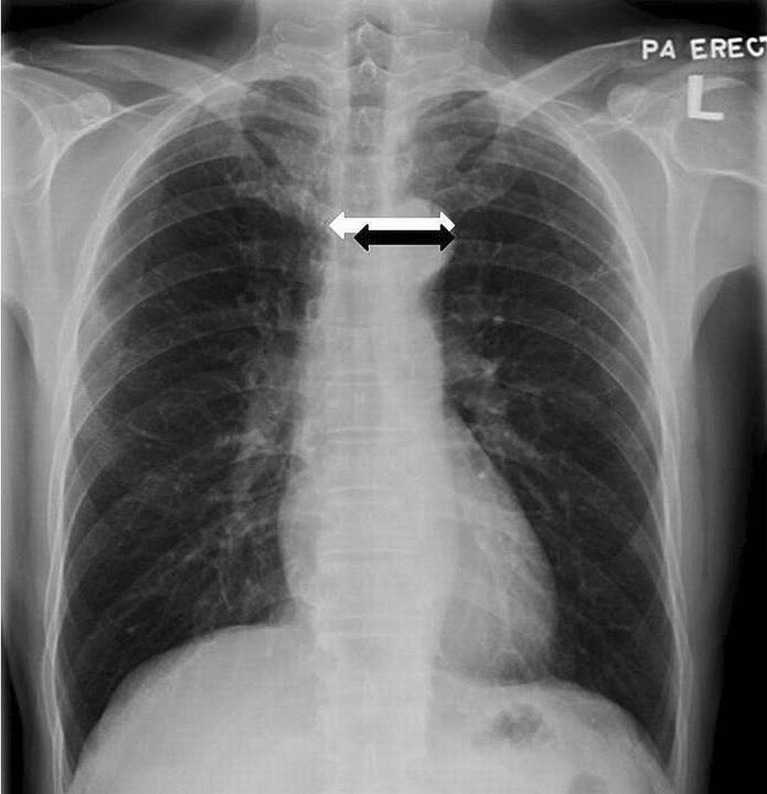
PA chest radiograph showing mediastinal measurement: MW (*white arrow*) and LMW (*black arrow*)

All chest radiographs from 220 subjects were reviewed by two independent radiologists blinded to the clinical data and study group. The following parameters were measured, as shown in Fig. 1: (1) maximal MW as defined by maximal distance from the right lateral border to the left lateral border of the superior mediastinum at the level of the aortic knob and (2) maximal LMW as defined by the maximal distance taken from midline of the trachea to left lateral border of the mediastinum at the level of the aortic knob. The MWR, defined as the ratio of LMW and MW, was thus calculated. In order to standardize measurement method and to minimize measurement error, no magnification was used and each parameter was measured three times by each individual radiologist with the mean value obtained. Discrepancy of measurement, if any, was resolved after subsequent consensus.

### Statistical analysis

Interobserver agreement for the measured data was evaluated and expressed with the *κ* statistic, which was excellent with the kappa value (*κ*) >0.90 for all MW and LMW measurements on both PA and AP chest radiographs. Statistical analyses were then performed by SPSS 16.0 for Windows (SPSS Inc.) separately for both PA and AP films with the use of independent sample *t* test. Differences with a *p* value of <0.05 were considered to be statistically significant. Receiver operating characteristic (ROC) curves were then generated with the cutoff values of the respective parameters determined from the coordinates along the curves to accommodate 100 % sensitivity and best diagnostic accuracy for each criterion.

## Results

**Fig. 2:**
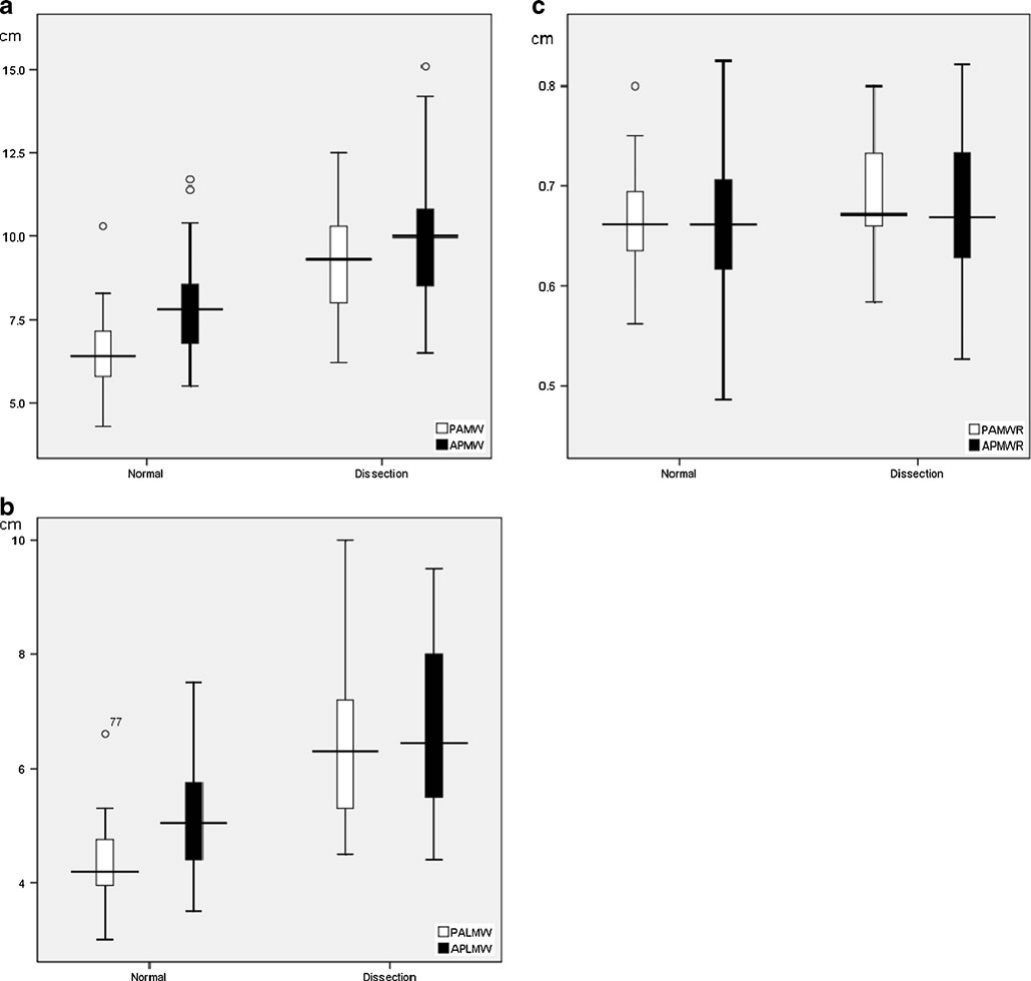
Box plot showing the distribution of PA and AP chest radiographic **a** MW, **b** LMW, and **c** MWR between normal and dissection groups

Radiographs from both the dissection and normal groups were matched for age, patient positioning, and radiographic technique with no statistically significant difference so that a nonbiased comparison could be achieved. The distribution of MW, LMW, and MWR for both dissection and normal groups with regard to AP and PA chest radiographs were represented by box plots in Fig. 2a–c, respectively.

For PA chest radiograph, the mean MW was 9.28 ± 1.67 cm in the dissection group and 6.45 ± 0.95 cm in the normal group; the mean LMW was 6.40 ± 1.44 cm in the dissection group and 4.30 ± 0.59 cm in the normal group; and the mean MWR was 0.687 ± 0.056 in the dissection group and 0.665 ± 0.045 in the normal group. MW and LMW were statistically significantly higher in the dissection group than in the normal group (MW and LMW: *p* < 0.001), while MWR between the two groups also reached a statistically significant difference (*p* = 0.022).

**Fig. 3:**
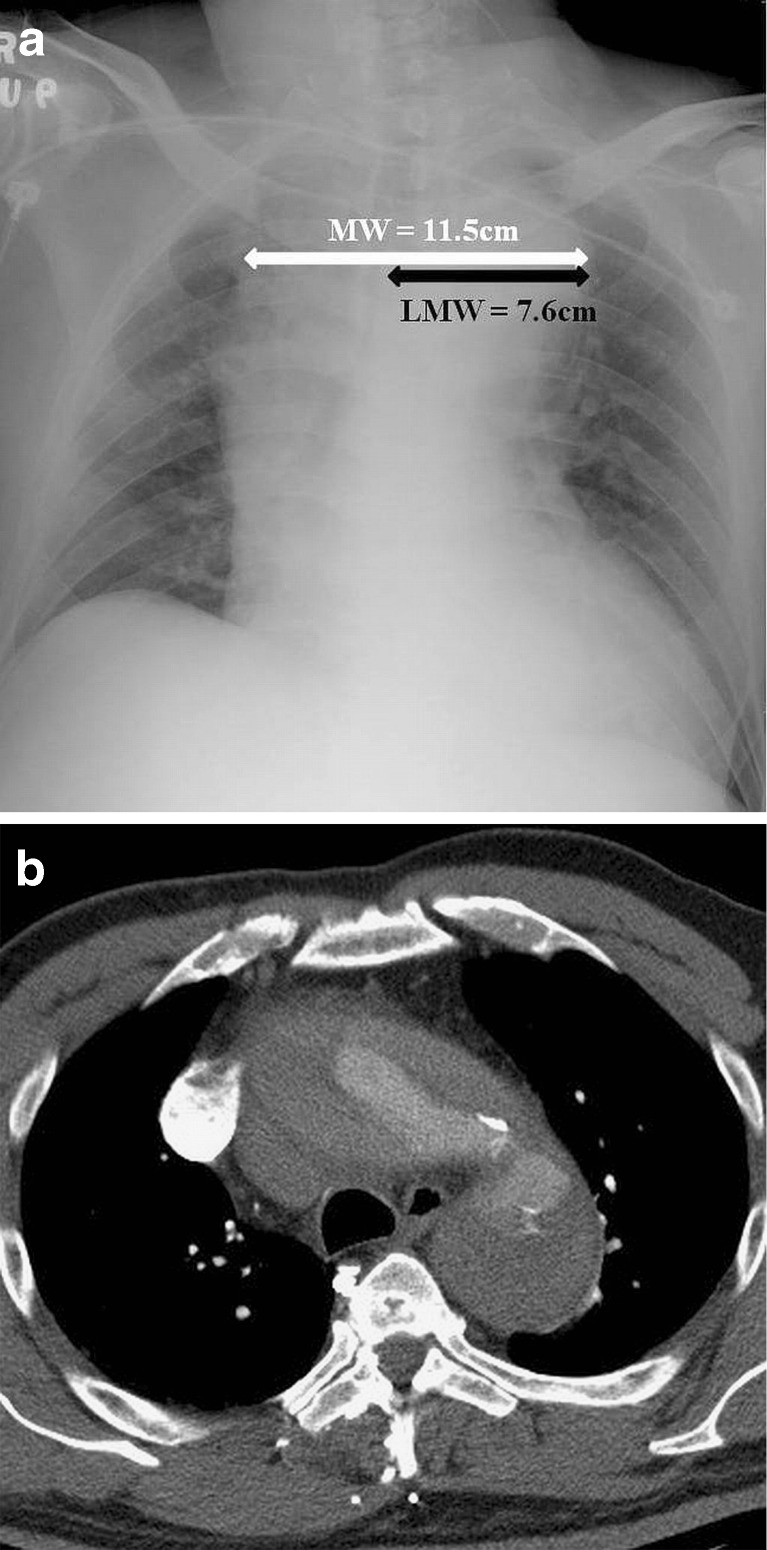
Acute type A aortic dissection in a 46-year-old man. **a** AP chest radiograph showing marked widening of the mediastinum with MW and LMW measuring 11.5 and 7.6 cm, respectively. **b** Corresponding selected image of CT aortogram confirms type A aortic dissection

For the AP chest radiograph, the mean MW was 9.76 ± 1.72 cm in the dissection group and 7.85 ± 1.29 cm in the normal group; the mean LMW was 6.61 ± 1.32 cm in the dissection group and 5.13 ± 0.89 cm in the normal group; and the mean MWR was 0.678 ± 0.078 in the dissection group and 0.656 ± 0.070 in the normal group. MW and LMW were statistically significantly higher in the dissection group than in the normal group (*p* < 0.001), while MWR did not reach statistical significance however (*p* = 0.117). A representative example is shown in Fig. 3a, b.

**Fig. 4:**
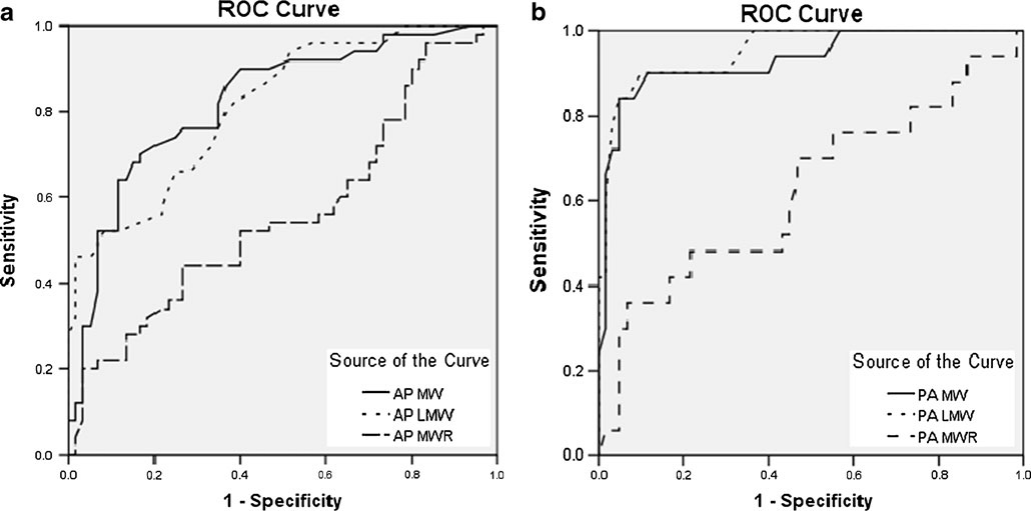
ROC curves for different measured variables in **a** AP and **b** PA chest radiographs

All corresponding values did not demonstrate any statistical significance in direct comparison between PA and AP groups. According to the ROC curve analysis (Fig. 4a), LMW was the most powerful parameter on PA chest radiograph, with an area under the curve of 0.952. The respective cutoff values to achieve 100 % sensitivity were LMW = 4.45 cm and MW = 6.15 cm but carried low specificities of 63.3 and 43.3 %, respectively, only. The optimal cutoff values in balancing high sensitivity and specificity were LMW = 4.95 cm (sensitivity, 90 %; specificity, 90 %; positive predictive value, 88.2 %; negative predictive value, 91.5 %) and MW = 7.45 cm (sensitivity, 90 %; specificity, 88.3 %; positive predictive value, 86.5 %; negative predictive value, 91.4 %). For AP chest radiographs, MW was the most powerful parameter, carrying an area under the curve of 0.823 as determined by the ROC curves (Fig. 4b). The respective cutoff values to achieve 100 % sensitivity were LMW = 4.35 cm and MW = 6.40 cm but associated with extremely low specificities of 22.7 and 6.7 %, respectively. The optimal cutoff values were LMW = 5.45 cm (sensitivity, 76 %; specificity, 65 %; positive predictive value, 64.4 %; negative predictive value, 76.4 %) and MW = 8.65 cm (sensitivity, 72 %; specificity, 80 %; positive predictive value, 75.0 %; negative predictive value, 77.4 %). These were summarized in Tables 1 and 2.

**Table 1:** Comparison of optimal cutoff values of MW and LMW for best diagnostic power between PA and AP chest radiographs

	PA	AP
MW (cm)	7.45	8.65
	Sn, 90 %; Sp, 88.3 %	Sn, 72 %; Sp, 80 %
	PPV, 86.5 %; NPV, 91.4 %	PPV, 75.0 %; NPV, 77.4 %
LMW (cm)	4.95	5.45
	Sn, 90 %; Sp, 90 %	Sn, 76 %; Sp, 65 %
	PPV, 88.2 %; NPV, 91.5 %	PPV, 64.4 %; NPV, 76.4 %

*MW* mediastinal width, *LMW* left mediastinal width, *Sn* sensitivity, *Sp* specificity, *PPV* positive predictive value, *NPV* negative predictive value

**Table 2:** Comparison of cutoff values of MW and LMW to achieve 100 % sensitivity between PA and AP chest radiographs

	PA	AP
MW (cm)	6.15	6.40
	Sp, 43.3 %	Sp, 6.7 %
LMW (cm)	4.45	4.35
	Sp, 63.3 %	Sp, 22.7 %

*MW* mediastinal width, *LMW* left mediastinal width, *Sp* specificity

## Discussion

Aortic dissection is not an infrequent entity, with a reported incidence of 5–10 out of 1,000,000 per year [13]. Yet patients often present with nonspecific clinical symptoms raising diagnostic difficulty. Much had been mentioned regarding the diagnostic accuracy of various findings on chest radiograph as indicator of thoracic aortic rupture in blunt chest trauma. These included widening of the mediastinum, blurring of the aortic knob or aortic knob width, tracheal shifting to the right, left apical cap, depression of the left mainstem bronchus below 40°, widened left paraspinal line, abnormal MW–chest width ratio, hemothorax, pneumothorax, and rib fractures [14–18]. MW >8 cm had been a widely used screening tool for aortic rupture [19–21]. However, only little emphasis was made on acute nontraumatic aortic rupture and aortic dissection. This can be important because differences exist between the two groups with regard to their etiology, age group, site of aortic injury, clinical course, and progression. In addition, the accentuation effect of AP film on MW as opposed to PA film can cause potential measurement error [22, 23]. The use of LMW and MWR had been proposed to minimize such accentuation effect on AP film [11], but its feasibility in acute nontraumatic aortic dissection has yet to be tested.

Our results demonstrated that only minor differences existed between PA and AP chest radiographs regarding the cutoff values of MW (PA, 6.15 cm; AP, 6.40 cm) and LMW (PA, 4.45 cm; AP, 4.35 cm) in achieving 100 % sensitivity, but with a tradeoff of low specificity which became worse in AP projection. Considerable differences were observed when the cutoff values were defined at the optimal level with best diagnostic accuracy according to ROC curves: MW (PA, 7.45 cm; AP, 8.65 cm) and LMW (PA, 4.95 cm; AP, 5.45 cm). MW was found to be the most powerful radiographic parameter on AP chest radiograph, while LMW was the most powerful on PA chest radiograph. In contrary, MWR was less useful and seemed unreliable, which could be related to artificial group differences produced from the mathematical relation. Overall, PA projection was significantly more accurate than AP projection, achieving high sensitivity and specificity. This could be explained by the considerable wide range or variability of the measured parameters with significant overlapping between normal and dissection groups in AP chest radiograph (as shown in Fig. 2) compared with those taken from PA chest radiograph. Therefore, the use of LMW alone in PA chest radiograph would provide a more accurate prediction in the assessment of acute aortic dissection, while a combination of LMW and MW would allow improved accuracy in determining acute aortic dissection in AP chest radiograph.

A major limitation was the variability of radiographic quality and view that might have influenced accurate assessment and measurement of the parameters. This would be particularly problematic with the AP chest radiographic technique as it might be difficult to maintain the same FFD or at its desirable distance for all subjects. Perhaps this could be the major reason behind its overall inferior diagnostic accuracy with significantly low specificity. In addition, the presence of aortic unfolding or age-related variation in aortic contour in the elderly would result in widening of the mediastinum which would also be accentuated in AP projection, hence leading to diagnostic dilemma with a false-positive result that could not be accounted for even with the use of LMW. On the contrary, results from PA chest radiograph demonstrated consistent excellent diagnostic accuracy with high sensitivity and specificity.

Variation in sites of aortic injury in cases with nontraumatic aortic dissection can give rise to a false-negative result in mediastinal widening. While traumatic aortic injury classically occurs at the level of aortic isthmus, the location of nontraumatic aortic dissection is more variable [24]. Type A aortic dissection will readily result in mediastinal widening, while type B aortic dissection may or may not lead to mediastinal widening. Similarly, those presenting with acute aortic rupture and mediastinal hemorrhage will also result in significant mediastinal widening as opposed to those without rupture but with aortic widening only from intramural hematoma. Indeed, due to the complexity and wide spectrum of changes in nontraumatic aortic dissection, its radiographic features are more variable and difficult to predict compared with traumatic aortic rupture. Our preliminary results can be used as reference to act as radiographic screening tool in clinical practice. Further larger-sized study will be helpful to validate our preliminary results, in particular, to address the difference of impact between type A and type B aortic dissection on mediastinal widening. Nevertheless, one should remember that management should still be made according to the American College of Radiology (ACR) criteria [25], in that the primary role of chest radiography is to rule out other thoracic pathology since up to 20 % of patients with aortic dissection may have normal chest radiographs. Therefore, any recognizable radiographic evidence of aortic disorder or even normal-appearing chest radiograph in patients with suspected nontraumatic aortic dissection should prompt immediate evaluation to establish or exclude such diagnosis.

## Conclusion

MW measurement is a useful radiographic screening tool for nontraumatic aortic dissection, showing higher diagnostic accuracy with PA projection. It will be reasonable to have a low threshold for proceeding to CT aortogram in patients with suspected nontraumatic aortic dissection, especially in the elderly group of patients as well as in patients with widened mediastinum on AP chest radiograph, while a negative PA chest radiograph may provide a higher negative predictive value, hence suggestion of normal aorta. Nevertheless, management should still be based on the ACR criteria and remains on clinical ground/suspicion.
